# Can routine data be used to estimate the mental health service use of children and young people living on Gypsy and Traveller sites in Wales? A feasibility study

**DOI:** 10.1371/journal.pone.0281504

**Published:** 2023-02-17

**Authors:** Sarah Rees, Richard Fry, Jason Davies, Ann John, Louise Condon

**Affiliations:** 1 Population Data Science, Swansea University Medical School, Swansea, United Kingdom; 2 School of Psychology, Swansea University, Swansea, United Kingdom; 3 School of Health and Social Care, Swansea University, Swansea, United Kingdom; University of New Mexico Health Sciences Center, UNITED STATES

## Abstract

**Introduction:**

Gypsies and Travellers have poorer physical and mental health than the general population, but little is known about mental health service use by Gypsy and Traveller children and young people. Finding this group in routine electronic health data is challenging, due to limited recording of ethnicity. We assessed the feasibility of using geographical markers combined with linked routine datasets to estimate the mental health service use of children and young people living on Traveller sites.

**Methods:**

Welsh Government supplied a list of Traveller site postcodes included in Caravan Counts between 2012 and 2020. Using spatial filtering with data from the Adolescent Mental Health Data Platform (ADP) at Swansea University’s SAIL Databank, we created a cohort of Traveller site residents aged 11–25 years old, 2010–2019. ADP algorithms were used to describe health service use, and to estimate incidence and prevalence of common mental disorders (CMD) and self-harm.

**Results:**

Our study found a subgroup of young Gypsies and Travellers (n = 802). We found no significant differences between our cohort and the general population for rates of CMD or self-harm. The rate of non-attendance for psychiatric outpatient follow-up appointments was significantly higher in our cohort. Rates were higher (but not statistically significant) among Gypsies and Travellers for measures suggesting less well-managed care, including emergency department attendance and prescribed CMD medication without follow-up. The small size of the cohort resulted in imprecise estimates with wide confidence intervals, compared with those for the general population.

**Conclusions:**

Gypsies and Travellers are under-represented in routine health datasets, even using geographical markers, which find only those resident in authorised traveller sites. Routine data is increasingly relied upon for needs assessment and service planning, which has policy and practice implications for this underserved group. To address health inequalities effort is required to ensure that health datasets accurately capture ethnicity.

## Introduction

Gypsies and Irish Travellers are recognised as distinct ethnic groups in the UK under the 2010 Equality Act [1]; they share a cultural, social and political identity with Roma people, who are the largest ethnic minority group in Europe [2–4]. In the UK the term ’Roma’ specifically denotes people from continental Europe with a Gypsy background [5]. However the European meaning of ’Roma’ is far broader, and "while it includes Gypsies and Irish Travellers, is not the way in which most British communities would identify themselves" [6]. ’Gypsy’ remains the predominant term used by people in the UK to self-identify their ethnicity; see for example the website of Friends, Families and Travellers, a traveller-led charity [7] and guidance for journalists and editors produced by the media organisation The Travellers’ Times [8]. Official UK policy uses this terminology, and refers to Gypsies and Travellers as distinct from Roma; this is reflected in data collection tools such as the UK census, which since 2011 has included ’Gypsy or Irish Traveller’ as an ethnic group, and since 2021 has featured a ’Roma’ ethnic group distinct from ’Gypsy or Irish Traveller’ [9].

According to the 2011 census, approximately 55,000 people in England and 3,000 in Wales (around 0.1% of the total populations of both nations) identified their ethnicity as Gypsy or Irish Traveller [10,11], although this is likely to be a significant under-estimate of the true population [6]. Roma people worldwide are subject to severe socio-economic inequalities [12] and have poorer physical and mental health than majority populations and other ethnic minority groups [13–17]. Self-reported mental health is worse among Roma populations in continental Europe [18], Ireland [19] and the UK [20]. Discrimination and social exclusion [21,22] exacerbate mental health problems and suicide rates are disproportionately high among travellers [23,24]. Gypsies, Roma and Travellers experience barriers to accessing health services, which are greater for conditions such as mental illness which are particularly stigmatised in this group [17,22].

Roma children have a higher prevalence of health risk factors [16,25,26], accompanied by lower uptake of preventive health services [17,27]. To date there has been little exploration of their mental health needs. A Slovakian survey found no difference in mental health difficulties between Roma and non-Roma children [28], whereas a study of children in Romania and Bulgaria, based on data derived from the School Children Mental Health Study in Europe (SCHME) [29], found Roma children to be at higher risk of conditions including anxiety, depression and conduct disorder [18]. An Irish survey described young Travellers as using negative terminology to refer to mental health difficulties and sharing beliefs that people in their ethnic group ‘suffer in silence’ rather than access services [30].

In the UK, routine data (such as those collected when individuals access health services) are increasingly used to inform policy and practice [31,32]. Analysis of routine health data from NHS services in Wales is an established method used to describe patterns of mental illness and self-harm among both the general population and in children and young people [33–38]. However, information about the health and social care needs of Gypsies and Travellers is hard to find in routinely collected data [39,40]. Despite being a protected characteristic under the 2010 Equality Act [1], and being included as an ethnic group in the 2011 Census of England and Wales, Gypsy and Traveller ethnicity is not recorded within routine national NHS data in England [6]. Prior to 2017 there was not a distinct code for Gypsy and Traveller ethnicity in Welsh health data [41], which limits the availability of historic data from Wales. At the time of writing, the extent to which the new Gypsy and Traveller ethnicity code has been implemented in Wales is not clear [42], and the volume of currently available data is insufficient for a study of this type.

Outside the UK, place-based data linkages have been used to identify people of Roma ethnicity within routine data sets. In Hungary around one-quarter of Roma people live in segregated Roma settlements, enabling this geographical marker to be used to interrogate national health insurance records and provide data on healthcare utilisation and premature death [43]. Similarly, a study used attendance at exclusively-Roma schools in Romania and Bulgaria as a marker of children’s ethnicity [18]. In the UK around a quarter of Gypsies and Travellers live in a caravan or other mobile or temporary structure [10], meaning that postcode data for officially recognised Gypsy and Traveller sites provides an opportunity for the flagging of Gypsies and Travellers in health datasets [44]. The Welsh Government holds data on Gypsy and Traveller sites in Wales, including site postcode, to inform the biannual Caravan Count [45].

The aims of this study were;

- to assess the feasibility of using Welsh routine health data in conjunction with Welsh Caravan Count postcode information to create a cohort of children and young people who, according to the address history in their GP record, have lived in a Traveller site while under the age of 18 (defined as a site included in the Welsh Government Biannual Caravan Count);
- to describe the use of NHS primary and secondary care services for common mental disorders (CMD; anxiety and depression), and for self-harm in the study cohort.

## Methods

### Study design

This was a retrospective population-based electronic cohort study.

### Data sources

The study was conducted using routine National Health Service (NHS) data from Wales, housed in the Adolescent Mental Health Data Platform (ADP) [46], a specialist resource bringing together routine datasets to support research examining the mental health of children and young people, while preserving their anonymity. This study used assets developed by the ADP, including code lists defining conditions of interest, and algorithms to estimate measures such as incidence and prevalence rates.

The ADP holds data from multiple sources; this study used data obtained from the SAIL Databank, which is a repository of health-related routine data from Wales [47,48]. The SAIL datasets are linkable due to the creation of an Anonymous Linkage Field (ALF) which uniquely identifies an individual, while preserving anonymity, and is based on matching the NHS number or other demographic features in each dataset with records in the Welsh Demographic Service Dataset. This dataset comprises details of all individuals who have been resident in Wales and registered with a Welsh GP, and acts as a population spine for Wales [49]. We used the Welsh Demographic Service Dataset to create the study cohort, and then linked this cohort to the Welsh Longitudinal General Practice dataset for events in primary care, the Patient Episode Dataset for Wales for elective and emergency inpatient admissions, the Outpatient Appointment Dataset for secondary care outpatient appointments, the Emergency Department Dataset for emergency department attendances and the Annual District Deaths Extract to identify date of death. Metadata for all datasets is available online [50].

The Welsh Government carries out a biannual Gypsy and Traveller Caravan Count, a census of caravans located in both authorised sites (those with planning permission or other status preventing planning enforcement action) and unauthorised sites (those without planning permission) [51]. Authorised sites include those socially rented (operated by local authorities or registered social landlords), and also privately funded sites (those rented via the private sector) [51]. Underpinning the caravan count is information regarding the locations of all known Gypsy and Traveller sites in Wales, including (where possible) the site postcode. The most recent caravan count (at the time of writing) was carried out in January 2020 [51]. We obtained from Welsh Government a list of postcodes designating all the Gypsy and Traveller sites in Wales which have featured in the caravan count since 2012. Using the location of these postcodes, we applied Geographic Information Systems methods to extract Unique Property Reference Numbers (UPRN) found within the spatial proximity of Gypsy and Traveller sites. The postcode-UPRN extraction method developed as part of this study potentially overestimates the number of potential residences available in each postcode. Postcodes, by definition, are a delivery mechanism for postal services and do not necessarily pertain to particular types of housing or different subgroups of the population. To resolve this we attached to each UPRN a housing type indicator derived from Address Base Premium [52]; this indicator distinguished UPRNs referring to houses or flats from those referring to caravans. Each UPRN was converted into a Residential Anonymous Linkage Field (RALF), which is used as a linkage key field in SAIL data to uniquely refer to a property, and which can be used in conjunction with the ALF to identify residents of each property [53].

We defined two levels of Gypsy and Traveller site, to allow for sensitivity analysis; the strict definition included only those RALFs with a housing type of ‘caravan’. The broad definition included RALFs with a housing type of ‘caravan’, and also RALFs where the housing type was ‘unknown’ and the site type was ‘authorised’. We used the RALF identifiers to anonymously link individual level health datasets to potential residential locations at each Gypsy and Traveller site across Wales, based on ALFs with a residence period in a Gypsy and Traveller site RALF.

### Study population and setting

We created a cohort of all children and young people registered with a SAIL-supplying GP practice in Wales who had their 11^th^– 25^th^ birthdays between 1^st^ January 2010 and 31^st^ December 2019. The exact birth date is not available in SAIL datasets, to preserve anonymity, so instead we used the week of birth to ascertain age. The cohort was created using a cleaned version of the Welsh Demographic Service Dataset [54], which is currently the only dataset in SAIL with a linked residential location (based on the RALF); this meant that only individuals registered with a GP in Wales at some point during the study period could be included in the cohort; individuals registered with GPs other than in Wales, or not registered with a GP, could not be detected. Data collection for each individual started on the latest of GP registration (plus six months for the analysis of primary care data), first day of 11^th^ birthday year or study start date. Data collection ended on the earliest of GP deregistration, last day of 25^th^ birthday year, death or study end. We included only those periods of time where an individual was registered with a SAIL-supplying GP practice; 324 out of 404 (80%) of practices in Wales are currently contributing data to SAIL. We used data collection start and end dates to derive person-years at risk (PYAR); this was the period during which an individual was at risk of an anxiety, depression or self-harm event appearing in their patient history. For events in primary care, we excluded data recorded in the first six months of registration at practice, to ensure we did not incorrectly record prevalent cases as new, where individuals move to a new practice and have their history re-recorded [35]. For every individual, we created a row of data for each year they were present in the data and met the study criteria. This resulted in ten annual cohorts comprising individuals reaching their 11^th^– 25^th^ birthdays for each year between 2010 and 2019.

### Measures

For each row of annual data we recorded sex, age and Welsh Index of Multiple Deprivation (WIMD) 2014 quintile, (which is an area-based composite measure of relative deprivation, based on the Lower Layer Super Output Areas defined following the 2011 census) [55], at last day of data collection in the given year (or nearest non-null record for WIMD). Individuals with null or contradictory values for sex and age were excluded. Records for which we were unable to identify a WIMD were recorded as “not known” and analysed as a distinct group. We used the list of Gypsy and Traveller site RALFs to create a binary variable flagging individuals within the cohort with at least one day of residence in a Gypsy and Traveller site RALF during childhood (when aged between 0 and 17; this residence period did not have to be during the study period); this was our exposure variable. We created two levels of Gypsy and Traveller site RALF flag, to allow comparison between the strict and broad definitions of a Gypsy and Traveller site.

We extracted a complete event history from primary care, emergency department, outpatient and inpatient datasets for each individual in the cohort, and flagged conditions of interest occurring during the study period, based on Read v2 codes [56], ICD10 codes [57] and Welsh national standard codes [58]. Conditions of interest were anxiety and depression, grouped together as Common Mental Disorders (CMD) [59,60], prescription of medication for CMD [33,35], which were present in the primary care dataset only, and self-harm [38,61]. We included codes for both diagnoses and symptoms in our definition of CMD. We were not able to be precise regarding CMD in emergency department and outpatient appointment datasets, as the clinical coding was not sufficiently detailed to allow identification of a specific diagnosis. Therefore in emergency department data we flagged attendance where there was a generic diagnosis code of ‘psychological/psychiatric condition’ and in outpatient appointment data we flagged appointments under any psychiatric speciality [62]. We defined self-harm as any code indicating intentional self-injury or self-poisoning regardless of motivation or suicidal intent and also included events of undetermined intention; we excluded self-harm where the sole diagnosis related to alcohol consumption or intoxication, in the absence of another code designating self-harm [63]. Self-harm was not recorded in outpatient data, so we were limited to data from primary care (using Read codes), emergency department (using Welsh standard codes for attendances relating to injury or poisoning due to self-harm or of undetermined intention) and inpatients (using ICD10 codes) to identify these events.

In emergency department data, we excluded follow-up attendances; in outpatient data we excluded follow-up appointments in the measures of incidence and prevalence but included them when estimating the proportion of the cohort with an appointment in the specialty of interest, and when estimating rates of non-attendance. Case definitions are summarised in Table 1. Lists of Read codes and ICD10 codes are available in S1 File.

**Table 1:** Case definitions.

Condition	Dataset full name	Case definition
Common Mental Disorders (CMD) and prescription for CMD (CMD MED)	Welsh Longitudinal General Practice Dataset (WLGP)	CMD: presence in patient history of a Read v2 code for diagnosis or symptoms of anxiety or depression [59,60] CMD MED: presence in patient history of a Read v2 code for prescription of antidepressant, anxiolytic or hypnotic medication [33,35]
	Patient Episode Dataset for Wales–inpatient admissions (PEDW)	CMD: presence in patient history of an ICD10 code in any episode/position for diagnosis or symptoms of anxiety or depression [60]
Psychological/ psychiatric events	Emergency Department Dataset (EDDS)	presence in patient history of an attendance with diagnosis code in any coding position = 21Z (Wales standard code for psychological/psychiatric conditions in emergency department) or F% (ICD10 codes for mental and behavioural disorders) [62]
	Outpatient Appointments Dataset (OPDW)	presence in patient history of a record of appointment attendance under Treatment Function Code = 7% (psychiatric specialties) [62]
Self-harm	WLGP	presence in patient history of a Read v2 code for deliberate self-harm or self-injury of undetermined intent, excluding self-harm solely by alcohol, with no other injury or poisoning [38,61]
	EDDS	presence in patient history of an attendance with Attend Group Code = 13 (‘self-harm’) or Attend Group Code = 14 (‘undetermined intent’) and (Diagnosis Code in any position = 10B-10Z (poisoning, excluding alcohol) or laceration to wrist of forearm) [38]
	OPDW	Not recorded
	PEDW	presence in patient history of an ICD10 code in any episode/position for deliberate self-harm or self-injury of undetermined intent, excluding self-harm by alcohol, with no other injury or poisoning [38]

### Statistical analysis

We described the composition of the study cohort, comparing the broad and strict cohorts with the general population, stratified by sex, and by age and WIMD 2014 quintile at the end of each year; counts were of PYAR rather than individuals. We estimated the observed unadjusted proportion of individuals having contacts with each service (at any time within the study period) for any reason, and also for contacts relating to CMD, (attendance for any psychological/psychiatric condition for emergency department and outpatient), prescription of a CMD medication; (primary care data only) and self-harm, stratified by sex where counts were sufficient.

Psychological therapy should be the first line treatment for anxiety in children and young people [33] and antidepressant medication should be prescribed to children only in combination with psychological therapy [64]. To examine this we compared the observed unadjusted proportion of individuals in the Gypsy and Traveller cohort and the general population who had a CMD medication event but no other service contact recorded in their history (within the study period); we carried out a sensitivity analysis in which we included outpatient non-attendances and cancellations, and not just appointments that were attended.

Outpatient non-attendance (DNA) rates are higher for psychiatric specialties than others, and higher non-attendance rates are associated with greater deprivation [62]. We included a measure comparing psychiatric outpatient non-attendance (DNA) rates in the Gypsy and Traveller cohort compared with the general population. 95% confidence intervals for binomial proportions, using the Wilson method [65], were calculated for all proportions.

We carried out Poisson regression, using Quasi-Poisson regression to account for over-dispersion [66], to derive rate ratios (RR) comparing the number of individuals in each of our cohorts with no service contact during the study period other than a CMD medication event, adjusted for sex, age (defined as age at the follow-up period midpoint for each individual) and WIMD 2014 (at start of follow-up). We carried out Quasi-Poisson analysis for annual DNA rates, adjusting for sex, and age and WIMD at each year end.

We estimated annual observed unadjusted rates per 1,000 PYAR for annual incidence (event in the calendar year which is the first in 12 or more months, but not necessarily the first ever event) and annual prevalence (event in the calendar year, regardless of whether it was the first or subsequent event) for each condition and in each dataset, for each year in the study period (except for emergency department data, for which we used the period 2011–2019, as this dataset was implemented during 2009, so we did not have a full year of data for identification previous events in 2010 [38]). We used PYAR as the denominator for incidence and prevalence measures, to account for individuals entering or leaving the cohort part-way through a year, because they either aged into or out of the cohort, arrived at or left a SAIL GP practice or died. Observed unadjusted incidence and prevalence rates for the Gypsy and Traveller cohort were compared with those in the general population (the non-Gypsy and Traveller cohort), stratified by sex where counts were sufficient. We carried out Poisson regression to estimate incidence rate ratios (IRR) comparing annual rates for the Gypsy and Traveller cohort and the non-Gypsy and Traveller cohort, adjusting for sex, age and WIMD 2014 quintile, using Quasi-Poisson regression [66] to account for over-dispersion.

Alpha for level of significance was set at P<0.05. Data preparation and analysis was carried out using IBM DB2 and R v4.0.2. All sections of the study are reported according to the REporting of studies Conducted using Observational Routinely collected health Data (RECORD) statement [67].

## Results

### Gypsy and Traveller site in SAIL datasets

The Gypsy and Traveller site list provided by Welsh Government resulted in a list of 3086 RALFs, each relating to a distinct address. 2318 (75.1%) of these RALFs were present in SAIL.

A breakdown by site type is shown in Table 2. Of the 2318 RALFs present in SAIL, 344 (14.8%) were of the building type ‘caravan’ (the strict Gypsy and Traveller cohort definition) and a further 34 (1.5%) were housing type ‘not known’ and site type ‘authorised’ (the broad Gypsy and Traveller cohort definition). These groups of RALFs formed the criteria for the selection of the study cohorts, defined as Gypsy and Traveller sites for subsequent analyses. There were 820 ALFs associated with the 344 RALFs comprising the strict cohort (those residing in RALFs with a housing type of ‘Caravan’ and a further 168 ALFs associated with the additional 34 RALFs in the broad cohort definition (those residing in RALFs with a housing type of ‘not known’ and a site type of ‘unknown’).

**Table 2:** Gypsy and Traveller site RALFs present in SAIL.

Housing type	Site type	total RALFs	SAIL RALFs	% present in SAIL	cohort ALFs *
Caravan	Authorised private	209	82	39.2	62
Caravan	Authorised socially rented	393	254	64.6	752
Caravan	Unauthorised	78	8	10.3	6
Detached	Authorised private	538	445	82.7	
Detached	Authorised socially rented	97	74	76.3	
Detached	Unauthorised	381	329	86.4	
Self-Contained Flat	Authorised private	91	43	47.3	
Self-Contained Flat	Authorised socially rented	13	5	38.5	
Self-Contained Flat	Unauthorised	59	26	44.1	
Semi-Detached	Authorised private	227	199	87.7	
Semi-Detached	Authorised socially rented	22	17	77.3	
Semi-Detached	Unauthorised	187	167	89.3	
Terraced	Authorised private	344	320	93.0	
Terraced	Authorised socially rented	56	40	71.4	
Terraced	Unauthorised	197	170	86.3	
Not known	Authorised private	67	34	50.7	168
Not known	Authorised socially rented	24	20	83.3	
Not known	Unauthorised	103	85	82.5	
Total		3086	2318	75.1	988

* Total ALFs will exceed numbers in CONSORT, as categories are not mutually exclusive (e.g. an individual may have lived in more than one housing type).

Abbreviations: RALF–Residential Anonymous Linkage Field; ALF–Anonymous Linkage Field.

### Study population

**Fig 1:**
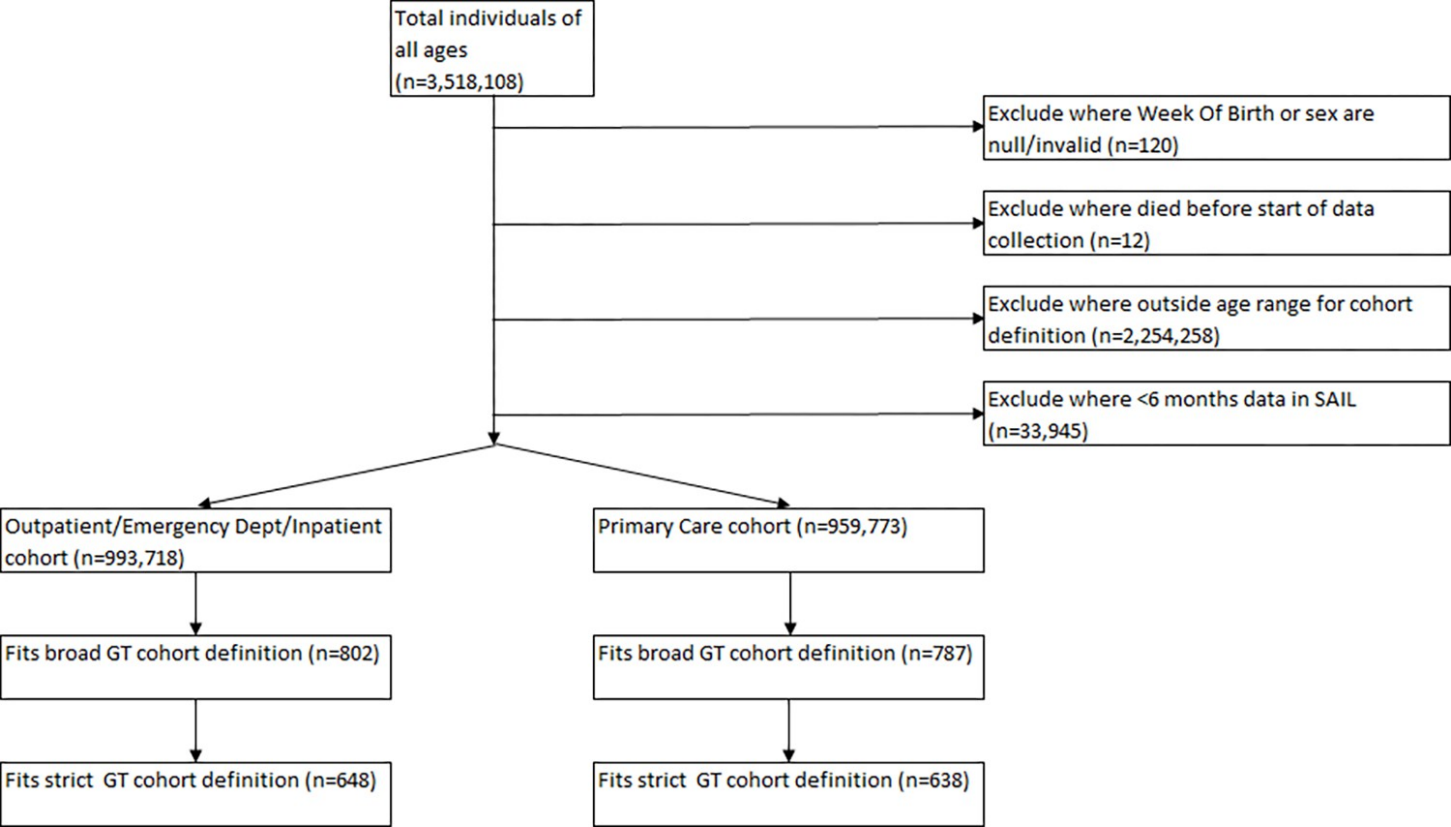
CONSORT diagram of study cohort.

The total study population (including Gypsy and Traveller and non-Gypsy and Traveller cohorts) comprised 993,718 individuals aged 11–25 between 2010 and 2019, contributing 4,680,819 person-years. We identified 648 individuals who fitted the strict Gypsy and Traveller cohort definition (contributing 3,531 person-years), and 802 who fitted the broad Gypsy and Traveller cohort definition, (contributing 4,281 person-years). For individuals in either the strict or the broad Gypsy and Traveller cohort, the mean total period of residence in an identifiable Gypsy and Traveller RALF, (including periods prior to the study start date, and up to 31^st^ Dec 2019) was 8.6 years (SD = 6.8 years). The study cohort composition is summarised in Fig 1.

**Fig 2:**
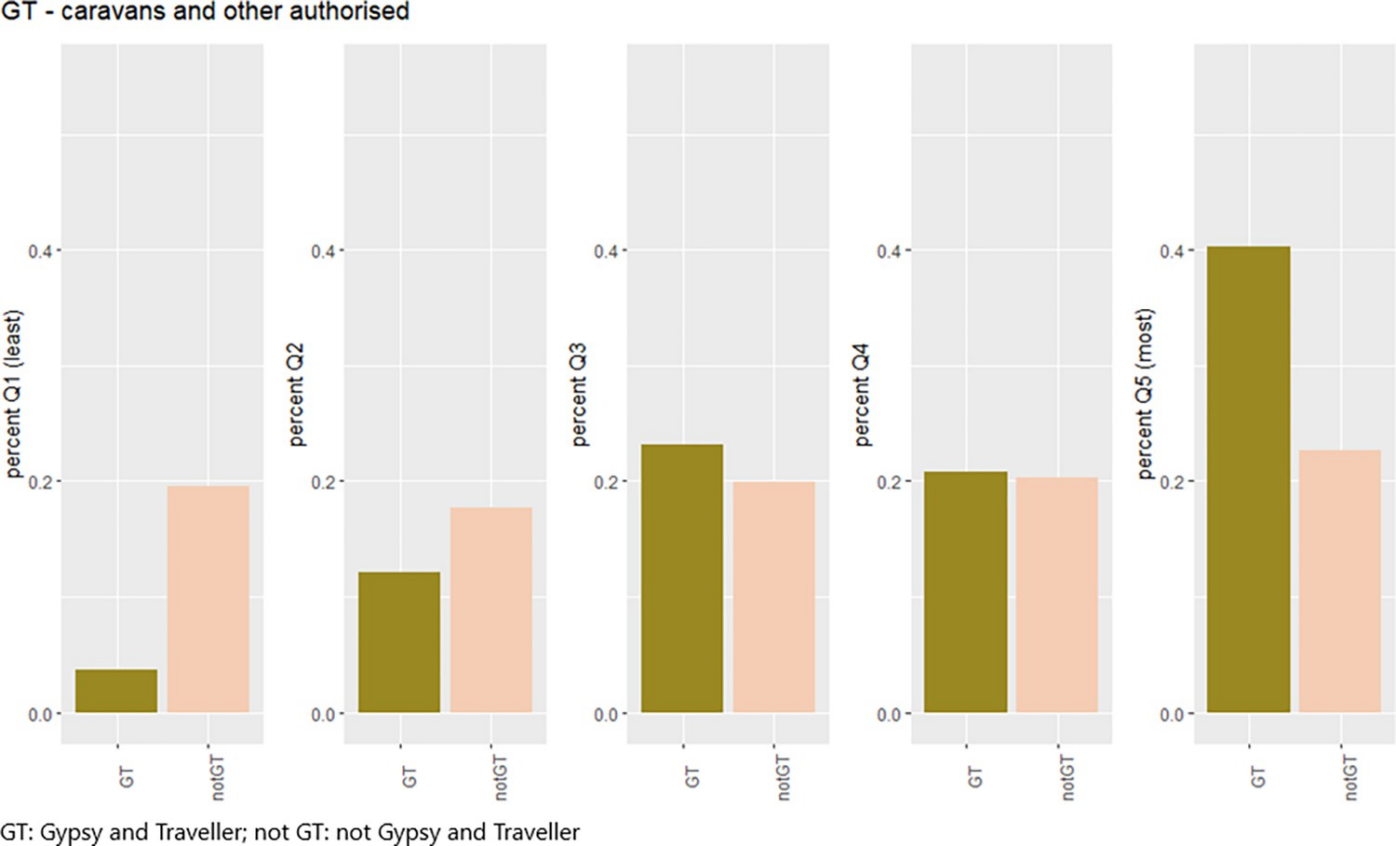
Study population; person-years by WIMD 2014 quintile.

The composition (in person-years) of the study cohort, comparing the strict and broad Gypsy and Traveller cohorts with the general population, is described by sex, year of birth and WIMD quintile in Table 3. The proportions by sex were similar in the non-Gypsy and Traveller cohort (49.0% male; 51.0% female) but in the Gypsy and Traveller cohorts there was a greater proportion of males than females (strict cohort = 56.4% male, 43.6% female; broad cohort = 55.9% male, 44.1% female). The age profile of both Gypsy and Traveller cohorts differed to that of the non-Gypsy and Traveller cohort, which had a greater proportion of older individuals; a comparison of the broad Gypsy and Traveller cohort and the non-Gypsy and Traveller cohort by age and year is shown in S1 Fig in S2 File. Mean age in both Gypsy and Traveller cohorts was 17.6 (SD = 4.3), and in the general population was 18.5 (SD = 4.3). The non-Gypsy and Traveller cohort was evenly distributed across WIMD 2014 quintiles; however this was not the case for the Gypsy and Traveller cohorts, where a greater proportion was in the most deprived WIMD 2014 quintile (44.7% of strict Gypsy and Traveller cohort; 40.3% of broad Gypsy and Traveller cohort; 22.3% of non-Gypsy and Traveller cohort) and a smaller proportion were in the least deprived quintile (2.9% of strict Gypsy and Traveller cohort; 3.7% of broad Gypsy and Traveller cohort; 19.2% of non-Gypsy and Traveller cohort). A comparison in the WIMD quintile composition of the broad Gypsy and Traveller cohort and the non-Gypsy and Traveller cohort is shown in Fig 2.

**Table 3:** Study population by study cohort, sex, age and deprivation quintile (counts are PYAR, not individuals).

		Case definition 1: caravans only				Case definition 2: caravans and authorised "not known"				Total cohort: *PYAR*
		NGTC: *PYAR*	GTC (strict definition): *PYAR*	NGTC: % of total cohort	GTC (strict definition): % of total cohort	NGTC: *PYAR*	GTC (broad definition): *PYAR*	NGTC: % of total cohort	GTC (broad definition): % of total cohort	
Total	-	4677293	3531	100.0	100.0	4676543	4281	100.0	100.0	4680824
Sex	Male	2387373	1993	51.0	56.4	2386974	2392	51.0	55.9	2389366
	Female	2289920	1538	49.0	43.6	2289569	1889	49.0	44.1	2291458
Age	11	276297	283	5.9	8.0	276247	333	5.9	7.8	276580
	12	275474	269	5.9	7.6	275422	321	5.9	7.5	275743
	13	275866	267	5.9	7.6	275814	319	5.9	7.5	276133
	14	278312	251	6.0	7.1	278263	300	6.0	7.0	278563
	15	280513	237	6.0	6.7	280461	289	6.0	6.8	280750
	16	284119	232	6.1	6.6	284069	282	6.1	6.6	284351
	17	289219	238	6.2	6.7	289169	288	6.2	6.7	289457
	18	301335	238	6.4	6.7	301284	289	6.4	6.8	301573
	19	325773	237	7.0	6.7	325723	287	7.0	6.7	326010
	20	347822	239	7.4	6.8	347774	287	7.4	6.7	348061
	21	354489	228	7.6	6.5	354436	281	7.6	6.6	354717
	22	353926	220	7.6	6.2	353871	275	7.6	6.4	354146
	23	349950	208	7.5	5.9	349901	257	7.5	6.0	350158
	24	344341	199	7.4	5.6	344299	241	7.4	5.6	344540
	25	339857	185	7.3	5.2	339810	232	7.3	5.4	340042
WIMD	1(least)	896126	101	19.2	2.9	896070	157	19.2	3.7	896227
quintile	2	809951	492	17.3	13.9	809927	516	17.3	12.1	810443
	3	912802	675	19.5	19.1	912483	994	19.5	23.2	913477
	4	931120	683	19.9	19.3	930914	889	19.9	20.8	931803
	5(most)	1042337	1580	22.3	44.7	1042192	1725	22.3	40.3	1043917
	NA	84957	-	1.8	0.0	84957	-	1.8	0.0	84957

Abbreviations: PYAR–Person Years At Risk; NGTC—Non Gypsy and Traveller Cohort; GTC–Gypsy and Traveller Cohort; WIMD–Welsh Index of Multiple Deprivation.

The number of individuals present in the data each year remained relatively stable for both sexes in the general population, while the number of males in the Gypsy and Traveller cohorts increased over the study period, with male counts in both the strict and broad Gypsy and Traveller cohorts peaking in 2017; a comparison of the broad Gypsy and Traveller cohort and the non-Gypsy and Traveller cohort is shown in S2 Fig in S2 File.

As the results of the descriptive analysis of the broad and strict cohorts were very similar, and presenting more detailed analysis for both cohorts would result in disclosure of small numbers by subtraction, all subsequent results refer to the broad cohort only.

### Observed unadjusted proportions with conditions of interest

**Fig 3:**
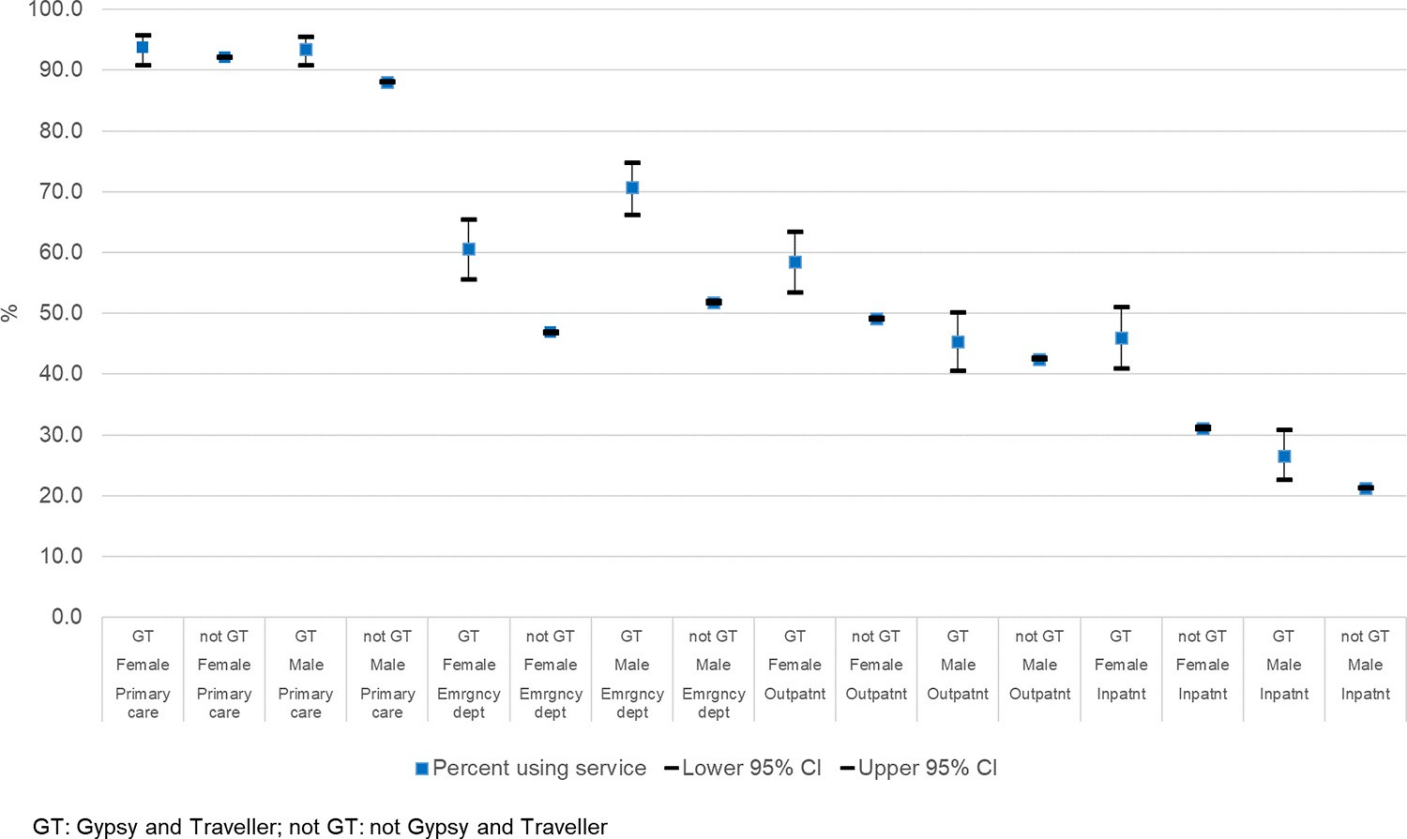
Observed unadjusted proportion with service contacts for any reason.

To establish overall usage of each service, we compared the observed unadjusted proportion of individuals in each cohort having service contacts during the study period for any clinical reason, stratified by sex; this is summarised in Fig 3. The proportion in contact with a service was significantly higher in the Gypsy and Traveller cohort than the non-Gypsy and Traveller cohort for both sexes in all services except for males in contact with outpatients and females in contact with primary care. The greatest difference was between attendances at the emergency department; 60.6% (95% CI 55.6–65.5%) of females and 70.7% (95% CI 66.2–74.8%) of males from the Gypsy and Traveller cohort had an attendance, compared with 46.9% (95% CI 46.8–47.0%) of females and 51.8% (95% CI 51.7–52.0%) of males in the non-Gypsy and Traveller cohort.

**Fig 4:**
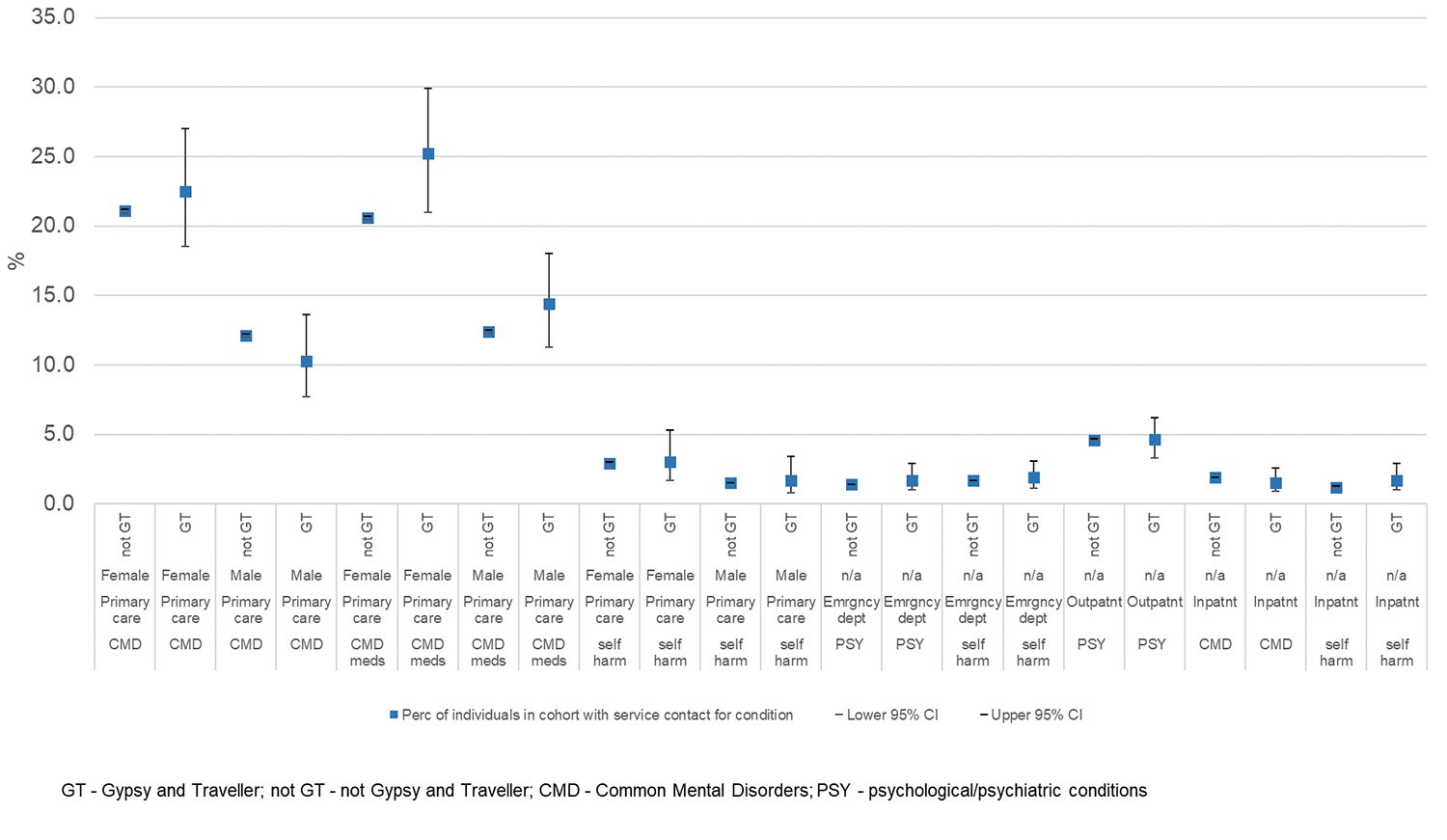
Observed unadjusted proportion with service contacts for conditions of interest.

Fig 4 summarises the proportion of individuals in each cohort with contacts with each service during the study period for the conditions of interest. As underlying counts were small in the Gypsy and Traveller cohort, it was not possible to split the results by sex for any data source other than primary care. Compared to the non-Gypsy and Traveller cohort, a greater (but non-significant) proportion of females from the Gypsy and Traveller cohort had a CMD event in primary care; (female Gypsy and Traveller cohort = 22.5%, 95% CI 18.5–27.0%; female non-Gypsy and Traveller cohort 21.1%, 95% CI 20.9–21.2%) and a significantly greater proportion of Gypsy and Traveller cohort females had a prescription for CMD medication in primary care; (female Gypsy and Traveller cohort = 25.2%, 95% CI 21.0–29.9%; female non-Gypsy and Traveller cohort = 20.6%, 95% CI 20.5–20.7%). A smaller (non-significant) proportion of Gypsy and Traveller cohort males than non-Gypsy and Traveller cohort males had a CMD event in primary care (male Gypsy and Traveller cohort = 10.3%, 95% CI 7.7–13.6%; male non-Gypsy and Traveller cohort = 12.1%, 95% CI 12.0–12.2%). Gypsy and Traveller cohort males had a greater (but non-significant) proportion of CMD medication events in primary care than non-Gypsy and Traveller cohort males (male Gypsy and Traveller cohort = 14.4%, 95% CI 11.3–18.0%; male non-Gypsy and Traveller cohort = 12.4%, 95% CI 12.3–12.5%). Proportions with a self-harm event in primary care were not significantly different between Gypsy and Traveller cohort and non-Gypsy and Traveller cohort in primary care.

Compared to males from the Gypsy and Traveller cohort, a significantly greater proportion of females had a CMD event in primary care (Gypsy and Traveller cohort female = 22.5%, 95% CI 18.5–27.0%; Gypsy and Traveller cohort male = 10.3%, 95% CI 7.7–13.6%) or a CMD medication event in primary care (Gypsy and Traveller cohort female = 25.2%, 95% CI 21.0–29.9%; Gypsy and Traveller cohort male = 14.4%, 95% CI 11.3–18.0%). Rates of self-harm in primary care were not significantly different for females from the Gypsy and Traveller cohort compared with males from the Gypsy and Traveller cohort. Rates of self-harm in primary care in the non-Gypsy and Traveller cohort were significantly higher for females than for males (non-Gypsy and Traveller cohort female = 2.9%, 95% CI 2.9–3.0%; non-Gypsy and Traveller cohort male = 1.5%, 95% CI 1.5–1.5%).

For all other data sources, we were not able to compare proportions by sex, as counts were too low in the Gypsy and Traveller cohort. There were no significant differences between the proportion of the Gypsy and Traveller cohort and non-Gypsy and Traveller cohorts with records in inpatient data for CMD or self-harm, in outpatient data for psychiatric specialties or in emergency department data for psychological/psychiatric conditions or self-harm.

### Rate of non-attendance at outpatients

Psychiatric outpatient non-attendance rates were compared between the Gypsy and Traveller cohort and non-Gypsy and Traveller cohort; observed unadjusted rates were estimated separately for new and follow-up appointments, as shown in Table 4. The difference in unadjusted non-attendance rate for new appointments was not significant (Gypsy and Traveller cohort = 23.2%, 95% CI 14.1–35.8%; non-Gypsy and Traveller cohort = 15.7%, 95% CI 15.4–16.0%), however the non-attendance rate for follow-up appointments was significantly higher in the Gypsy and Traveller cohort, compared with the non-Gypsy and Traveller cohort (Gypsy and Traveller cohort = 36.9%, 95% CI 30.0–44.4%; non-Gypsy and Traveller cohort = 15.1%, 95% CI 15.0–15.2%).

**Table 4:** Psychiatric outpatient DNA rates.

Cohort	Type of appointment	DNA: *n*	total attend + DNA: *n*	%	Lower CI	Upper CI
NGTC	New	11407	72646	15.7	15.4	16.0
NGTC	Follow Up	49157	326063	15.1	15.0	15.2
GTC	New	13	56	23.2	14.1	35.8
GTC	Follow Up	62	168	36.9	30.0	44.4

Abbreviations: NGTC—Non Gypsy and Traveller Cohort; GTC–Gypsy and Traveller Cohort; DNA–Outpatient non-attendance; CI = Confidence Interval.

Poisson regression, using Quasi-Poisson to account for over-dispersion [66], was run to derive rate ratios (RR) comparing non-attendance rates for the Gypsy and Traveller and non-Gypsy and Traveller cohorts, adjusted for sex, age and WIMD 2014 deprivation quintile. Results for new appointments were not significantly different (RR 1.13, 95% CI 0.21–3.32). Results for follow-up appointments were significantly higher for the Gypsy and Traveller cohort (RR 2.18, 95% CI 1.01–4.01).

### Proportion with prescription for CMD medication but no other service contact

We compared the unadjusted proportion of individuals in each cohort with a prescription for CMD medication in primary care but no record of any other service contact, as shown in Table 5. A significantly greater proportion of the Gypsy and Traveller cohort had a CMD medication event but no other service contact in their record (Gypsy and Traveller cohort = 5.7%, 95% CI 4.3–7.6%; non-Gypsy and Traveller cohort = 4.0%, 95% CI 4.0–4.0%). Results of a sensitivity analysis in which we included all outpatient appointments, including non-attendances and cancellations, and not just appointments attended, were identical.

**Table 5:** Individuals with prescription for anxiety or depression medication in primary care data, with no further service contacts.

Cohort	Contact for CMD MED in WLGP only: *n*	Total cohort	%	Lower CI	Upper CI
NGTC	38407	958986	4.0	4.0	4.0
GTC	45	787	5.7	4.3	7.6

Abbreviations: NGTC—Non Gypsy and Traveller Cohort; GTC–Gypsy and Traveller Cohort; WLGP–Primary Care dataset; CMD MED–prescription of medication with no further service contact; CI = Confidence Interval.

We ran a Quasi-Poisson regression to compare the proportion of each cohort with only a CMD medication event, adjusted for sex, age (at midpoint of follow-up period) and WIMD 2014 (at start of follow-up period). Once adjusted, the differences between Gypsy and Traveller cohort and non-Gypsy and Traveller cohort were no longer significant.

### Incidence and prevalence

Table 6 presents unadjusted annual incidence and prevalence rates per 1,000 PYAR for conditions of interest, separately for each data source, with 95% confidence intervals for Poisson rates. Suppression has been applied to counts where disclosure by differencing is possible between different measures. Counts in Table 6 refer to the total number of annual events that meet the definition for an incident or prevalent case in each category. As an individual can be an annual incident or an annual prevalent case in more than one year, this is not a count of individuals. We were able to report results for primary care stratified by sex, but counts were too low to stratify by age or WIMD quintile. We were able to report results from all other data sources by Gypsy and Traveller group only. We found no statistically significant differences between the Gypsy and Traveller cohort and the general population for annual incidence or prevalence rates in any data source.

**Table 6:** Unadjusted incidence and prevalence rates per 1,000 PYAR, by data source and condition group (for all data sources), and by sex (for primary care only).

Data source	Cond group	Sex	Cohort	Annual inc (n)	Annual inc rate	Annual inc lower CI	Annual inc upper CI	Annual prev (n)	Annual prev rate	Annual prev lower CI	Annual prev upper CI
WLGP	CMD	F	GTC	110	59.9	49.3	72.2	134	73.0	61.2	86.5
WLGP	CMD	F	NGTC	134914	61.4	61.0	61.7	167553	76.2	75.8	76.6
WLGP	CMD	M	GTC	57	24.3	18.4	31.5	71	30.2	23.6	38.2
WLGP	CMD	M	NGTC	71305	30.8	30.6	31.1	85177	36.8	36.6	37.1
WLGP	CMD MED	F	GTC	109	59.4	48.8	71.6	193	105.2	90.9	121.1
WLGP	CMD MED	F	NGTC	111830	50.9	50.6	51.2	216543	98.5	98.1	98.9
WLGP	CMD MED	M	GTC	65	27.7	21.4	35.3	118	50.3	41.6	60.2
WLGP	CMD MED	M	NGTC	64579	27.9	27.7	28.1	118276	51.1	50.8	51.4
WLGP	SH	F	GTC	<15	<8.2	<4.6	<13.5	15	8.2	4.6	13.5
WLGP	SH	F	NGTC	15951	7.3	7.1	7.4	17923	8.2	8.0	8.3
WLGP	SH	M	GTC	8	3.4	1.5	6.7	8	3.4	1.5	6.7
WLGP	SH	M	NGTC	7961	3.4	3.4	3.5	8632	3.7	3.7	3.8
PEDW	CMD	-	GTC	15	3.5	2.0	5.8	15	3.5	2.0	5.8
PEDW	CMD	-	NGTC	20850	4.5	4.4	4.5	22748	4.9	4.8	4.9
PEDW	SH	-	GTC	<18	<4.2	<2.5	<6.7	18	4.2	2.5	6.7
PEDW	SH	-	NGTC	13416	2.9	2.8	2.9	14660	3.1	3.1	3.2
OPDW	PSY	-	GTC	<37	<8.7	<6.1	<11.9	37	8.7	6.1	11.9
OPDW	PSY	-	NGTC	46089	9.9	9.8	10.0	50910	10.9	10.8	11.0
EDDS	PSY	-	GTC	<15	<3.9	<2.2	<6.4	15	3.9	2.2	6.4
EDDS	PSY	-	NGTC	14671	3.5	3.4	3.6	16029	3.8	3.8	3.9
EDDS	SH	-	GTC	<19	<4.9	<2.9	<7.6	19	4.9	2.9	7.6
EDDS	SH	-	NGTC	17777	4.2	4.2	4.3	19728	4.7	4.6	4.8

Abbreviations: WLGP–Primary Care/ dataset; PEDW–Inpatient Admission dataset; OPDW–Outpatient Appointment Dataset; EDDS–Emergency Department Dataset; F–Female; M–Male; NGTC—Non Gypsy and Traveller Cohort; GTC–Gypsy and Traveller Cohort; CMD MED–prescription of medication with no further service contact; SH–self harm; PSY–psychological or psychiatric conditions; inc–incidence; prev–prevalence; CI = Confidence Interval.

#### Comparison of unadjusted incidence and prevalence rates between males and females within the Gypsy and Traveller cohort: Primary care only

Compared with Gypsy and Traveller cohort males, Gypsy and Traveller cohort females had significantly higher rates of CMD and CMD medication in primary care across all measures (Gypsy and Traveller cohort CMD annual incidence: female = 59.9, 95% CI 49.3–72.2, male = 24.3, 95% CI 18.4–31.5; Gypsy and Traveller cohort CMD annual prevalence: female = 73.0, 95% CI 61.2–86.5; male = 30.2, 95% CI 23.6–38.2, Gypsy and Traveller cohort CMD medication annual incidence: female = 59.4, 95% CI 48.8–71.6, male = 27.7, 95% CI 21.4–35.3; Gypsy and Traveller cohort CMD medication annual prevalence: female = 105.2, 95% CI 90.9–121.1, male = 50.3, 95% CI 41.6–60.2). Suppression of small numbers prevented comparison of primary care self-harm annual incidence rates. There were no significant differences between Gypsy and Traveller cohort males and females for annual prevalence of self-harm, although rates were higher for females than males.

#### Adjusted incidence and prevalence rate ratios

A series of Quasi-Poisson regressions was run to derive IRRs comparing annual incidence and annual prevalence rates of the conditions of interest in the Gypsy and Traveller cohort compared with the non-Gypsy and Traveller cohort, adjusted for sex, age band and WIMD 2014 deprivation quintile, including an interaction between Gypsy and Traveller group and sex. Reference groups in all analyses were non-Gypsy and Traveller cohort, female sex, age band 11–14, and WIMD quintile 1 (least deprived). There were no significant differences between IRRs for Gypsy and Traveller cohort and non-Gypsy and Traveller cohort detected in any of the regression analyses. IRRs from regression results are shown in S1-S8 Tables S2 File.

**Fig 5:**
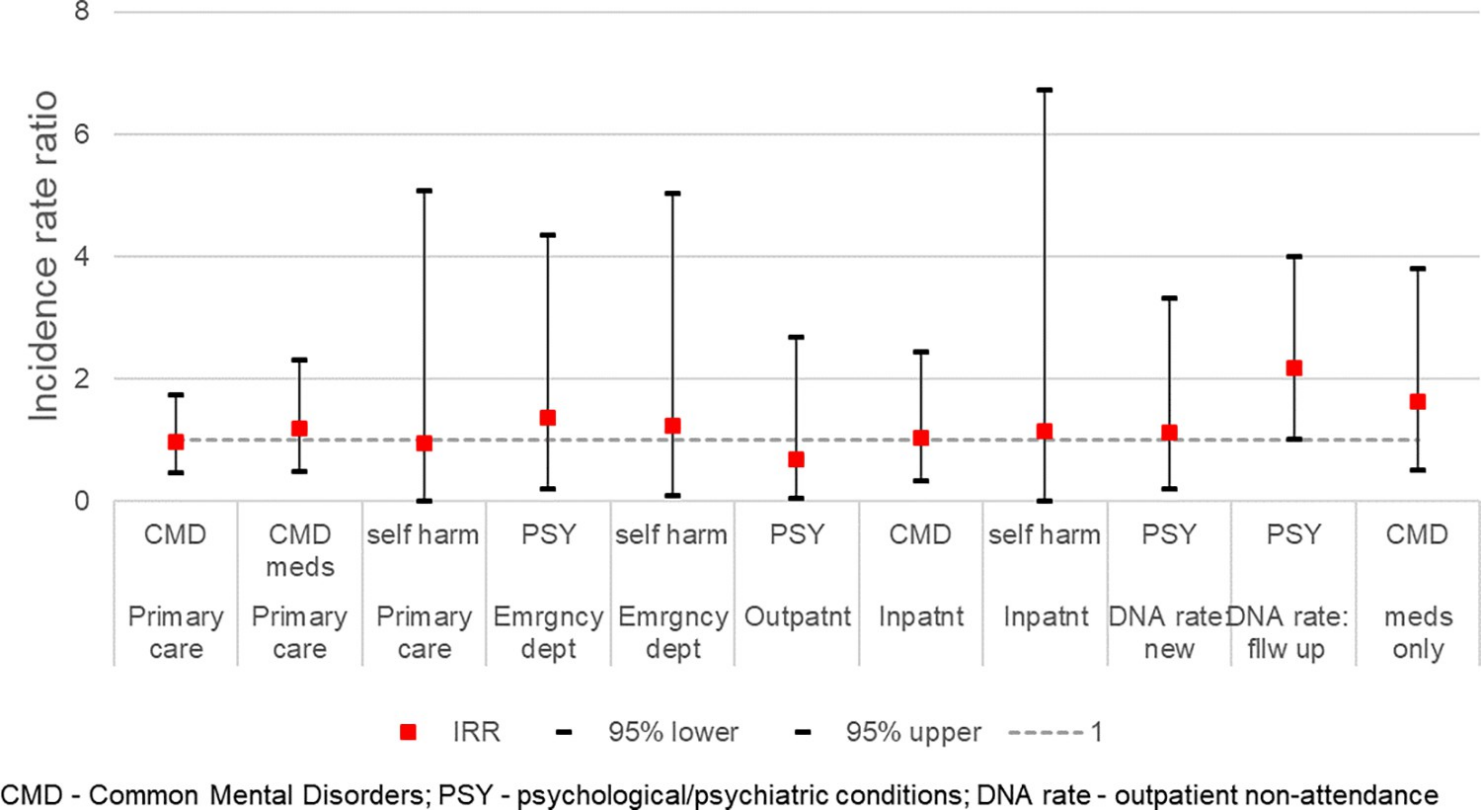
Annual incidence rate ratios for Gypsy and Traveller cohort compared with general population, adjusted for sex/age/WIMD 2014 quintile.

Fig 5 summarises the IRRs for all measures of annual incidence, and RRs for DNA rate and proportion with only a CMD medication event.

## Discussion

### Main findings in context of previous studies

This study explored the feasibility of using geospatial techniques in conjunction with routine health data to estimate service use for common mental disorders and self-harm, among children and young people from Gypsy and Traveller communities in Wales. A key feature of this approach is that it does not require Gypsies and Travellers to self-identify. This can be a significant obstacle, as reluctance to disclose ethnicity, for fear of discrimination, has been noted [44]. Our approach is also not reliant upon ethnicity coding within routine datasets; the quality of ethnicity coding in general is often poor in routine data, and a study of English NHS datasets found they contained an under-representation of minority ethnic groups, greater inconsistency of ethnicity coding in minority ethnic groups, and an over-representation of non-specific “other” groups [68]. Although ‘Gypsy or Irish Traveller’ was included as an ethnic category in the 2011 census, this category has not historically been used in NHS health systems [6], although it has recently been introduced in Wales. Despite ongoing concerns about racial discrimination, Gypsy, Roma and Traveller participants in the UNITING study [69] identified ethnicity recording within health systems as a priority intervention to support tailored service provision [70]. This participatory research showed that identification within health records was highly acceptable, when carried out in combination with other interventions designed to increase trust between service users and providers [70].

A major finding of our study was that this hard to reach group cannot be easily identified in routine health data sets in Wales, even using geographical markers. This has implications for the health of both this group and for that of other vulnerable groups, as routine data is increasingly relied upon to assess health needs and plan services. Lack of visibility in routine data is particularly important, for both Gypsies and Travellers and for other underserved minority groups, when understood in the context of proposals to use administrative data as the primary source for the census in 2031 and beyond, with supplementary surveys used only where gaps in administrative data are identified [71].

We found that the proportion of individuals in contact with service for any reason was greater among the Gypsy and Traveller cohort than in the non-Gypsy and Traveller cohort in almost all settings. This was initially surprising given that access to healthcare is known to be poor among Gypsies and Travellers [6], and barriers to accessing services include the organisation, affordability and acceptability of health systems, and service-user attributes such as health literacy, culture and language [17,72]. This finding may be an artefact of our methodology. Our approach depends upon GP registration in order to identify both the study cohort and the comparison group, because at present, no other SAIL datasets include the necessary RALF variable. This means that we will exclude individuals not registered with a Welsh GP. It is therefore possible that our study population over-represents people in the poorest health whose ill health has necessitated registration with a GP practice. Conversely we may be missing individuals who, due to comparatively better health, have not pre-emptively registered with a GP. We do not know whether this will disproportionately affect Gypsies and Travellers. Future developments may see the RALF added to other routine health datasets in SAIL, which would help to partially mitigate this; in particular its presence in emergency department data would permit the flagging of individuals who give a Gypsy and Traveller site as their address when using this service, but who are not registered with a GP (although we may not be able to link these individuals to other datasets).

### Strengths and limitations

Combining the caravan count site postcode information with data from the ADP enabled us to anonymously identify a subset of Gypsy and Traveller children and young people, with some significant limitations. At present it is necessary to use such methods, as the code to record Gypsy and Traveller ethnicity in Welsh health data does not appear to have been fully implemented across all datasets and volumes of data recorded with the new Gypsy and Traveller ethnicity code are currently too low to permit this type of analysis; an equivalent code does not yet exist in routine English NHS datasets. We were able to anonymously identify only those individuals resident on an officially designated Gypsy and Traveller site; we were not able to flag Gypsies and Travellers living in permanent dwellings such as houses or flats or individual dwellings (typically caravans) which have not been captured in official address registers. Previous studies have encountered similar limitations; a study in Hungary used a residential geographical marker in conjunction with national health insurance records to provide data on healthcare utilisation and premature death [43]. This study was able to identify differences in health usage patterns among individuals living in Roma settlements; however it was unable to assess the health of Roma people not living in designated settlements. As well as excluding a significant proportion of the Gypsy and Traveller population in Wales, this limitation prevents our study from comparing rates of CMD and self-harm among those residing in Gypsy and Traveller sites, with those residing in bricks and mortar housing, or assessing whether living in a site offers any protective value for mental health due to ethnic density effects [73]. Further, we were unable to flag residents in unauthorised sites, as although these sites feature in the caravan count, they do not usually have postcodes, which is an essential data item in our method.

For RALF linkages to be resolved within SAIL a volunteered address supplied upon GP registration need to be subsequently assigned a UPRN using a post-hoc address matching process. Where an individual volunteers an address with details missing, or which does not match an officially recorded address, UPRN assignment will fail, resulting a lack of RALF upon which linkage can be performed. The spatial filtering method using a postcode is also likely to include non-Gypsy and Traveller dwellings; we posit that the unauthorised sites include a large number of detached properties due to lower residential dwelling density and therefore more space for unauthorised sites to become established. Following a sensitivity analysis, we decided to include in our case definition those RALFs where the housing type was ‘unknown’ and the site type was ‘authorised’. This was because although these sites have been officially recognised by a local authority, there are complex planning restrictions and laws pertaining to residing in caravans [74], which means residences within a site may not officially be classified as a ‘caravan’ in a local authority planning and addressing system (hence ‘unknown’), particularly where a site may be subject to planning appeals.

Given that the 2011 census is likely to significantly under-represent the true Gypsy and Traveller population of Wales [6], due to low literacy levels, mistrust of authority and failure of the census enumeration process to include all Traveller sites [17,75], it is difficult to estimate the true proportion of the Welsh Gypsy and Traveller population captured in this study, as we have no reliable population count with which to compare our cohort. We cannot therefore ascertain the degree to which our findings are generalizable to the wider Gypsy and Traveller population.

Finding a greater proportion of males than females in the Gypsy and Traveller cohort was unexpected; however a similar pattern was found among these age groups in results from the 2011 census [76]. Further studies could investigate possible causes for this finding, to ascertain whether this reflects genuine population composition, or if there are administrative issues relating to the recording of information, or barriers preventing access to services, leading to females being missed from sources of data, such as the census and health datasets.

Case ascertainment for the older cohort members is more difficult as it is dependent on earlier years of data; for the oldest in the cohort (those aged 25 in 2010), a period of residence on a Gypsy and Traveller site between 1985 and 2003 is required, but as we have Gypsy and Traveller site information from only the 2012 census onwards, we may be missing sites that closed prior to this date. We adjusted all analysis for age, which will mitigate this gap to some extent. GP registration rates may be lower for the Gypsy and Traveller cohort. This may result in over-estimation of incidence and prevalence rates, as the denominator will not represent the whole population of Gypsies and Travellers.

### Policy, research and practice implications

Gypsies and Travellers experience particularly high stigma and shame associated with mental illness and self-harm, perceive use of mental health services negatively, and may not identify with the concept of good mental health [77]. They may also have experienced discrimination when attempting to access health services [17]. We found a higher proportion of both males and females in the Gypsy and Traveller cohort had been in contact with most services (for any clinical reason), but because identification of our cohort depended on a GP registration, this may be due to lower overall GP registration rates in this group, although we were not able to assess this in our study. However in keeping with previous studies, when looking at overall health service usage (not focusing solely on CMD), we observed particularly high usage of emergency department services in the Gypsy and Traveller cohort; this has been noted elsewhere, and attributed to poor experiences when accessing other NHS services [6]. GP practices and other health care providers should ensure that services are made acceptable and accessible to Gypsies and Travellers, and acknowledge and remove administrative and cultural barriers for those who may fear or who have experienced discrimination, or do not have a permanent address. Methods for doing this have been suggested in previous empirical research [17,78].

Our study builds upon research which has shown that the prevalence of common mental disorders is significantly higher in Gypsy and Traveller communities, compared to the general population [14]. However the small size of our study cohort meant that the rates we identified were imprecise and resulted in wide confidence intervals, and we identified very few significant differences between the cohorts. We did observe that although non-significant, rates were lower in the Gypsy and Traveller cohort for measures suggesting better managed care, and higher in the Gypsy and Traveller cohort for measure suggesting less well managed care. Further research in this area may be worthwhile; future studies should examine the identification and management of common mental disorders and self-harm for Gypsy and Traveller children and young people across a range of different settings.

Non-attendance rates for follow-up outpatient appointments were significantly higher in the Gypsy and Traveller cohort. This should be a priority area for future research, in order to confirm this finding in a more representative population of Gypsies and Travellers, and to understand and address the factors that may support or obstruct attendance. In particular, as this study includes only residents of Gypsy and Traveller sites, further comparison should be carried out to establish whether greater non-attendance is also associated with Gypsies and Travellers living in other types of accommodation that may be more conveniently located and with better access to hospitals and transport, or whether it is specifically associated with residing in a Gypsy and Traveller site.

## Conclusion

This study has highlighted the importance of detailed and accurate recording of ethnicity in routine health data. We have shown it is feasible to use linked data to assess the needs of young people who have lived in Gypsy and Traveller sites. However, we know some subgroups of the population engage less with health services for a multitude of different reasons, and further work is needed to understand whether a bias exists in using electronic health records which are dependent on engagement with services for studying different subgroups of the wider population.

The most vulnerable people (e.g. Gypsies and Travellers, asylum seekers and refugees, homeless people, and sex workers) are often missed in data collection [44], resulting in a lack of evidence about their healthcare needs and service utilisation. This omission is a key barrier to accessing accurate data regarding the health needs of these populations [79]. Although we were able to find a subset of the Welsh Gypsy and Traveller population in routine health data, the degree to which this subset is representative of the wider Gypsy and Traveller population in Wales, including those not living in Gypsy and Traveller sites, is difficult to assess. Lack of visibility for Gypsies and Travellers in routine health and administrative datasets may present a significant barrier to recognition of need and provision of services, particularly in light of suggested changes to key sources of data used for commissioning, such as the UK census. Health service providers in Wales should ensure that plans to record Gypsy and Traveller ethnicity are fully implemented across all datasets.

Abbreviation definition

## Supporting information

S1 File Code lists to define depression and anxiety symptoms, depression and anxiety medication and self-harm using ICD-10 and Read v2 Codes. (XLSX)

S2 File S1 Fig: Study population by age. S2 Fig: Study population by sex. S1-S8 Tables: Results of Poisson regression.
(PDF)

## Abbreviations

ADDE: Annual District Deaths Extract
ADP: Adolescent Mental Health Data Platform
ALF: Anonymous Linkage Field—a code uniquely identifying one individual, allowing datasets to be linked
CMD: Common Mental Disorders (anxiety and depression
CMD MED: A prescription for CMD medication issued by the GP
DNA: Did Not Attend—non-attendance at Outpatients
EDDS: Emergency Department Dataset
ICD-10: International Classification of Diseases 10th Revision
IRR: Incidence Rate Ratio
NHS: National Health Service
OPDW: Outpatient Appointment Dataset
PEDW: Patient Episodes Dataset for Wales
PSY: Service contacts in either Outpatients or Emergency Department for psychological/psychiatric reasons
PYAR: Person Years At Risk
RALF: Residential Anonymous Linkage Field—a code uniquely identifying one place of residence
RR: Rate ratio
SAIL: Secure Anonymised Information Linkage
UPRN: Unique Property Reference Number
WIMD: Welsh Index of Multiple Deprivation
WLGP: Welsh Longitudinal General Practice dataset
