# Supplementary material for: Can routine data be used to estimate the mental health service use of children and young people living on Gypsy and Traveller sites in Wales? A feasibility study
Source: PLoS One. 2023 Feb 17;18(2):e0281504. doi: 10.1371/journal.pone.0281504 (PMC9937479; doi:10.1371/journal.pone.0281504)
Supplement: S2 File — S2 Fig: Study population by sex. S1-S8 Tables: Results of Poisson regression. (PDF) [file pone.0281504.s002.pdf]

Supplementary Figure S1: study population; person-years by age

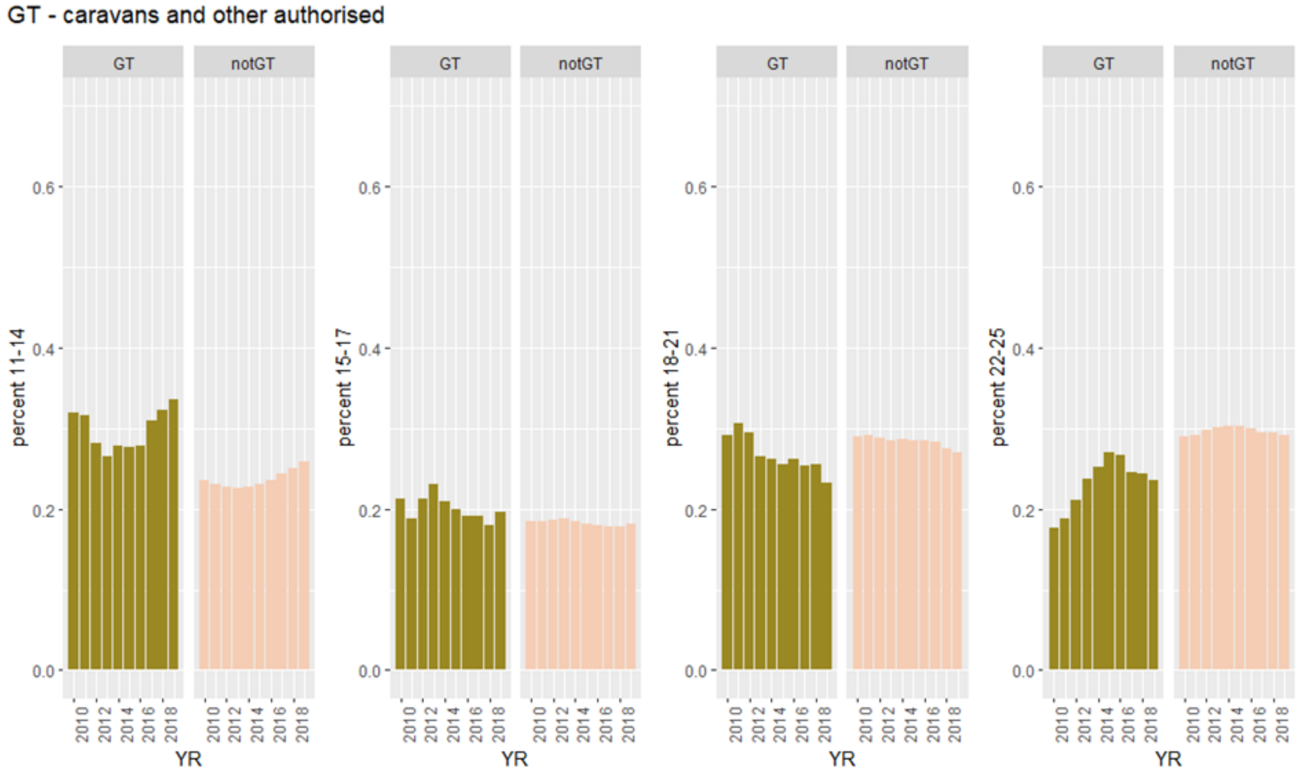

Supplementary Figure S2: study population; annual counts by sex

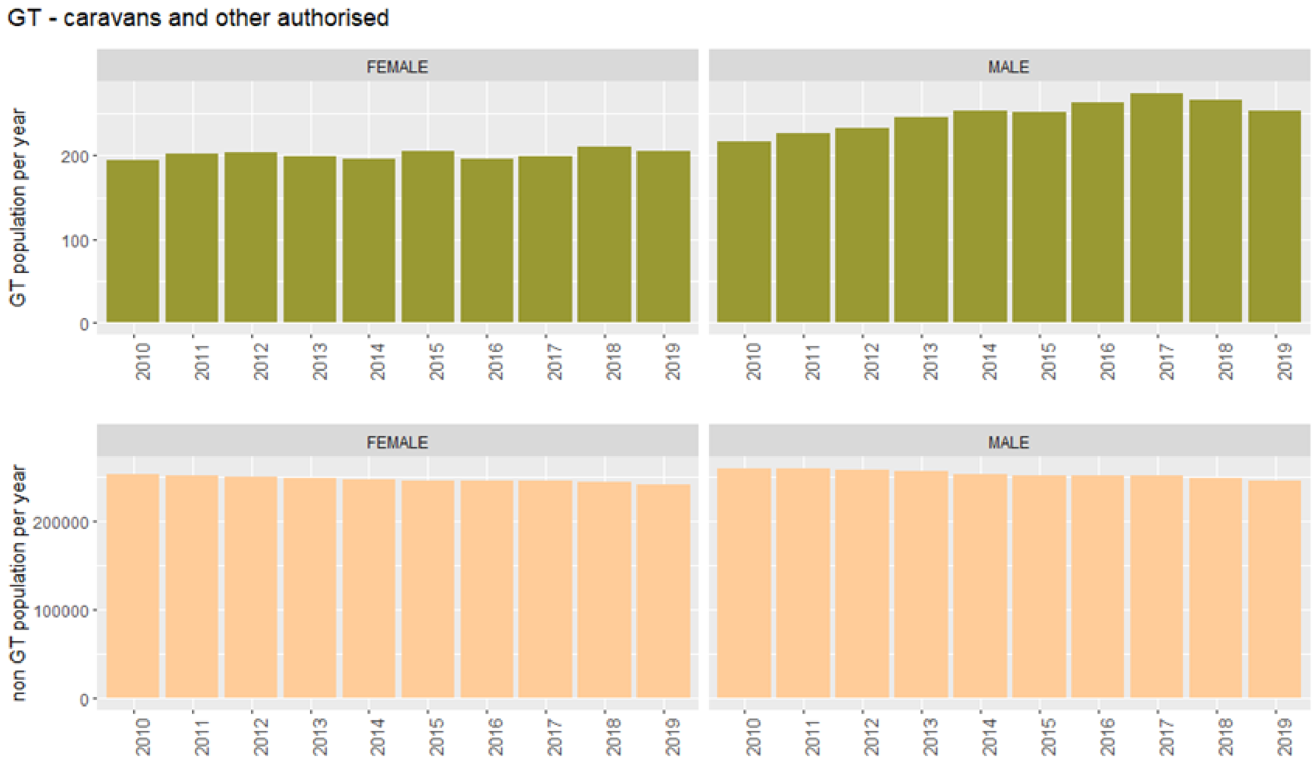

Key to tables 1-8: GTC - Gypsy and Traveller cohort; IRR – Incidence Rate Ratio; inc – incidence; prev – prevalence; CI – Confidence Interval

Reference groups for tables 1-8: Sex = female, Age = 11-14, WIMD 2014 quintile = 1 (least deprived)

Supplementary Table S1: Quasi-Poisson: CMD in WLGP

	annual inc IRR	annual inc lower CI	annual inc upper CI	annual prev IRR	annual prev lower CI	annual prev upper CI
(Intercept)	0.01	0.01	0.01	0.01	0.01	0.01
GTC	0.98	0.48	1.75	0.96	0.47	1.73
MALE	0.51	0.49	0.52	0.49	0.47	0.50
AGE 15-17	2.89	2.69	3.11	3.04	2.81	3.29
AGE 18-21	5.61	5.26	5.99	6.53	6.09	7.00
AGE 22-25	5.96	5.59	6.36	7.13	6.66	7.65
WIMD 2	1.07	1.01	1.12	1.08	1.02	1.14
WIMD 3	1.13	1.08	1.19	1.15	1.09	1.21
WIMD 4	1.31	1.25	1.38	1.34	1.28	1.41
WIMD 5 (most)	1.50	1.43	1.57	1.56	1.48	1.63
WIMD NA	0.47	0.39	0.56	0.48	0.40	0.58
GTC*MALE	0.80	0.24	2.28	0.84	0.26	2.44

GTC and GTC * MALE SEX not significant. All others except WIMD 2 (first ever incidence only) significant at P=<0.05

Supplementary Table S2: Quasi-Poisson: MED in WLGP

	annual inc IRR	annual inc lower CI	annual inc upper CI	annual prev IRR	annual prev lower CI	annual prev upper CI
(Intercept)	0.00	0.00	0.00	0.01	0.01	0.01
GTC	1.19	0.50	2.32	1.10	0.32	2.62
MALE	0.55	0.53	0.58	0.52	0.50	0.55
AGE 15-17	4.39	3.78	5.12	3.06	2.53	3.72
AGE 18-21	18.31	16.04	21.03	14.43	12.26	17.12
AGE 22-25	20.97	18.37	24.06	20.07	17.09	23.78
WIMD 2	1.09	1.02	1.17	1.08	0.98	1.18
WIMD 3	1.19	1.12	1.27	1.18	1.09	1.29
WIMD 4	1.43	1.34	1.52	1.43	1.31	1.55
WIMD 5 (most)	1.67	1.57	1.77	1.67	1.55	1.81
WIMD NA	0.40	0.30	0.50	0.44	0.32	0.60
GTC*MALE	0.83	0.22	2.77	0.90	0.14	4.63

GTC and GTC * MALE SEX not significant. All others (Except WIMD 2 for annual prevalence only) significant at P=<0.05

Supplementary Table S3: Quasi-Poisson: SH in WLGP

	annual inc IRR	annual inc lower CI	annual inc upper CI	annual pre IRR	annual prev lower CI	annual prev upper CI
(Intercept)	0.00	0.00	0.00	0.00	0.00	0.00
GTC	0.95	0.02	5.08	0.90	0.02	4.96
MALE	0.48	0.42	0.54	0.46	0.41	0.52
AGE 15-17	3.00	2.47	3.66	3.19	2.61	3.90
AGE 18-21	2.64	2.19	3.21	2.80	2.31	3.41
AGE 22-25	1.93	1.59	2.35	2.05	1.68	2.51
WIMD 2	1.23	0.99	1.52	1.23	1.00	1.53
WIMD 3	1.32	1.08	1.63	1.32	1.08	1.63
WIMD 4	1.71	1.41	2.08	1.71	1.41	2.08
WIMD 5 (most)	2.09	1.74	2.52	2.10	1.75	2.54
WIMD NA	0.46	0.17	0.97	0.45	0.16	0.96
GTC*MALE	0.93	0.00	64.37	0.90	0.00	71.38

GTC and GTC * MALE SEX not significant. All others except WIMD 2 and WIMD NA significant at P=<0.05

Supplementary Table S4: Quasi-Poisson: PSY in EDDS

	annual inc IRR	annual inc lower CI	annual inc upper CI	annual pre IRR	annual prev lower CI	annual prev upper CI
(Intercept)	0.00	0.00	0.00	0.00	0.00	0.00
GTC	1.36	0.21	4.35	1.35	0.20	4.37
MALE	0.77	0.71	0.83	0.75	0.69	0.81
AGE 15-17	2.92	2.52	3.39	3.07	2.64	3.59
AGE 18-21	3.41	2.98	3.93	3.62	3.14	4.18
AGE 22-25	2.82	2.46	3.25	3.04	2.64	3.52
WIMD 2	1.32	1.14	1.52	1.34	1.15	1.55
WIMD 3	1.54	1.34	1.77	1.55	1.34	1.78
WIMD 4	1.83	1.61	2.10	1.86	1.63	2.13
WIMD 5 (most)	2.07	1.82	2.35	2.11	1.85	2.40
WIMD NA	0.73	0.48	1.05	0.70	0.45	1.02
GTC*MALE	0.41	0.01	5.21	0.38	0.01	5.14

GT and GT&*SEX not significant. All others except WIMD NA significant at P=<0.05

Supplementary Table S5: Quasi-Poisson: SH in EDDS

	annual inc IRR	annual inc lower CI	annual inc upper CI	annual pre IRR	annual prev lower CI	annual prev upper CI
(Intercept)	0.00	0.00	0.00	0.00	0.00	0.00
GTC	1.23	0.09	5.04	1.37	0.12	5.37
MALE	0.73	0.66	0.80	0.70	0.64	0.77
AGE 15-17	3.79	3.14	4.59	4.01	3.29	4.91
AGE 18-21	3.83	3.21	4.61	4.10	3.40	4.98
AGE 22-25	2.90	2.41	3.50	3.13	2.58	3.81
WIMD 2	1.18	0.97	1.45	1.19	0.97	1.47
WIMD 3	1.50	1.24	1.80	1.51	1.25	1.83
WIMD 4	2.22	1.87	2.64	2.24	1.88	2.69
WIMD 5 (most)	2.79	2.37	3.30	2.84	2.40	3.37
WIMD NA	0.43	0.20	0.79	0.41	0.19	0.78
GTC*MALE	0.36	0.00	10.68	0.30	0.00	8.73

GT and GT&*SEX not significant. All others except WIMD 2 significant at P=<0.05

Supplementary Table S6: Quasi-Poisson: PSY in OPDW

	annual inc IRR	annual inc lower CI	annual inc upper CI	annual pre IRR	annual prev lower CI	annual prev upper CI
(Intercept)	0.01	0.01	0.01	0.01	0.01	0.01
GTC	0.69	0.06	2.68	0.74	0.07	2.82
MALE	0.89	0.83	0.95	0.88	0.82	0.94
AGE 15-17	1.59	1.46	1.74	1.64	1.50	1.80
AGE 18-21	0.81	0.74	0.88	0.84	0.77	0.93
AGE 22-25	0.58	0.52	0.64	0.60	0.55	0.67
WIMD 2	1.06	0.94	1.20	1.07	0.94	1.22
WIMD 3	1.22	1.08	1.37	1.23	1.09	1.39
WIMD 4	1.62	1.45	1.81	1.64	1.47	1.84
WIMD 5 (most)	1.84	1.66	2.05	1.89	1.70	2.10
WIMD NA	0.52	0.32	0.78	0.50	0.31	0.76
GTC*MALE	1.00	0.07	15.81	0.85	0.05	13.21

GT and GT&*SEX not significant. All others except WIMD 2 significant at P=<0.05

Supplementary Table S7: Quasi-Poisson: CMD in PEDW

variables	annual inc IRR	annual inc lower CI	annual inc upper CI	annual pre IRR	annual prev lower CI	annual prev upper CI
(Intercept)	0.00	0.00	0.00	0.00	0.00	0.00
GTC	1.05	0.33	2.44	0.96	0.27	2.35
MALE	0.35	0.33	0.37	0.34	0.32	0.36
AGE 15-17	3.07	2.70	3.51	3.21	2.79	3.69
AGE 18-21	5.72	5.09	6.45	5.95	5.25	6.76
AGE 22-25	7.40	6.60	8.33	7.86	6.96	8.91
WIMD 2	1.12	1.02	1.24	1.12	1.01	1.24
WIMD 3	1.28	1.16	1.40	1.29	1.17	1.42
WIMD 4	1.74	1.59	1.90	1.77	1.61	1.94
WIMD 5 (most)	2.26	2.08	2.46	2.31	2.12	2.53
WIMD NA	0.55	0.40	0.72	0.53	0.39	0.72
GTC*MALE	0.16	0.00	2.18	0.16	0.00	2.68

GTC and GTC * MALE SEX not significant. All others significant at P=<0.05

Supplementary Table S8: Quasi-Poisson: SH in PEDW

	annual inc IRR	annual inc lower CI	annual inc upper CI	annual pre IRR	annual prev lower CI	annual prev upper CI
(Intercept)	0.00	0.00	0.00	0.00	0.00	0.00
GTC	1.16	0.02	6.71	1.36	0.04	7.10
MALE	0.44	0.38	0.51	0.42	0.36	0.48
AGE 15-17	3.35	2.68	4.22	3.55	2.83	4.48
AGE 18-21	2.17	1.74	2.74	2.23	1.78	2.82
AGE 22-25	1.65	1.31	2.10	1.72	1.36	2.18
WIMD 2	1.25	0.96	1.62	1.24	0.96	1.62
WIMD 3	1.36	1.06	1.74	1.35	1.05	1.73
WIMD 4	1.74	1.38	2.21	1.74	1.38	2.21
WIMD 5 (most)	2.18	1.75	2.73	2.18	1.75	2.73
WIMD NA	0.62	0.24	1.29	0.60	0.23	1.26
GTC*MALE	0.90	0.00	92.98	0.72	0.00	52.61

GTC and GTC * MALE SEX not significant. All others except WIMD 2 and WIMD NA significant at P=<0.05
